# Metabolic modeling of denitrification in *Agrobacterium tumefaciens*: a tool to study inhibiting and activating compounds for the denitrification pathway

**DOI:** 10.3389/fmicb.2012.00370

**Published:** 2012-10-18

**Authors:** Marlies J. Kampschreur, Robbert Kleerebezem, Cristian Picioreanu, Lars Bakken, Linda Bergaust, Simon de Vries, Mike S. M. Jetten, Mark C. M. van Loosdrecht

**Affiliations:** ^1^Department of Biotechnology, Delft University of TechnologyDelft, Netherlands; ^2^Department of Plant and Environmental Sciences, Norwegian University of Life SciencesǺs, Norway; ^3^Department of Chemistry, Biotechnology, and Food Sciences, Norwegian University of Life SciencesǺs, Norway; ^4^Department of Microbiology, Radboud University NijmegenIWWR, Nijmegen, Netherlands

**Keywords:** *Agrobacterium tumefaciens*, denitrification, metabolic model, nitric oxide, nitrite reduction, nitrous oxide, NO reduction

## Abstract

A metabolic network model for facultative denitrification was developed based on experimental data obtained with *Agrobacterium tumefaciens*. The model includes kinetic regulation at the enzyme level and transcription regulation at the enzyme synthesis level. The objective of this work was to study the key factors regulating the metabolic response of the denitrification pathway to transition from oxic to anoxic respiration and to find parameter values for the biological processes that were modeled. The metabolic model was used to test hypotheses that were formulated based on the experimental results and offers a structured look on the processes that occur in the cell during transition in respiration. The main phenomena that were modeled are the inhibition of the cytochrome c oxidase by nitric oxide (NO) and the (indirect) inhibition of oxygen on the denitrification enzymes. The activation of transcription of nitrite reductase and NO reductase by their respective substrates were hypothesized. The general assumption that nitrite and NO reduction are controlled interdependently to prevent NO accumulation does not hold for *A. tumefaciens.* The metabolic network model was demonstrated to be a useful tool for unraveling the different factors involved in the complex response of *A. tumefaciens* to highly dynamic environmental conditions.

## Introduction

Denitrification is an important process in the global nitrogen cycle, which is investigated at many levels ranging from gene expression to global nitrogen fluxes. Denitrification is mostly performed by facultative denitrifiers, which reduce oxygen when available and switch to nitrate or nitrite after oxygen depletion. During denitrification nitrogen oxides are used as electron acceptor by an electron transport chain similar to that used in aerobic respiration (Zumft, 1997). A complete denitrification pathway comprises four subsequent steps: nitrate reduction, nitrite reduction, nitric oxide (NO) reduction and nitrous oxide (N_2_O) reduction. Each reduction step is catalyzed by a specific reductase enzyme.

Incomplete denitrification can give rise to emission of NO and N_2_O (e.g., Schuster and Conrad, 1992). Emission occurs when intermediates accumulate because the electron fluxes over the four subsequent denitrification steps are unbalanced or when incomplete pathways are present or expressed in denitrifying organisms (e.g., Ferguson, 1994). The emission of NO is unwanted as it is toxic while N_2_O is a potent greenhouse gas (Houghton et al., 2001) and dominant ozone-depleting substance (Ravishankara et al., 2009). A better understanding of the regulatory network of denitrifying bacteria may lead to development of strategies to prevent NO and N_2_O emission.

In metabolism, several levels of organization can be distinguished: the genome, transcriptome, proteome and metabolome. Cells regulate their metabolism in response to changes in their environment. While the genome is more or less constant, the transcriptome, proteome and metabolome respond to the actual environmental conditions. Current advances in molecular biology lead to a great increase in availability of genome sequences and transcriptomic, proteomic and metabolomic data. Consequently, there is a high demand for analysis of the generated data and use these data for a broader understanding of metabolic regulation, for which a metabolic model can be a good tool.

Induction of the denitrification pathway after oxygen depletion is regulated by multiple promoters for gene expression. Even though the promoters are comparable in many types of bacteria, the exact regulation mechanism depends on the type of micro-organism (e.g., Rodionov et al., 2005). Gene expression responds to specific environmental conditions like oxygen and nitrogen oxides concentrations, and possibly other compounds like metal ions (Zumft, 1997). Many individual relationships between promoters and specific genes have been identified that play a role in the regulation of the denitrification process, but only limited information is available on the main factors governing the overall response during transition from aerobic to anoxic conditions. To characterize such overall response patterns, Bergaust et al. (2008) conducted batch experiments with *A. tumefaciens*. *A. tumefaciens* is a facultative denitrifying micro-organism. *A. tumefaciens* contains the genes for periplasmic dissimilatory nitrate reductase (*nap*), nitrite reductase (*nirK*), and NO reductase (*norB*), but it lacks the genes encoding N_2_O reductase (Wood et al., 2001). As a consequence, its final product of denitrification is N_2_O.

Bergaust et al. monitored the response of *A. tumefaciens* to transition from aerobic to anoxic conditions in terms of substrate uptake and intermediate and end-product accumulation. Additionally, gene expression profiles of key enzymes were measured during the aerobic-anoxic transition. The results demonstrated unbalanced expression of denitrification enzymes under certain conditions, resulting in uncontrolled accumulation of NO. Despite the rigorous experimental approach, the authors were not able to find a coherent explanation for all the observations made, and the need for a formalized model-based analysis of the experiments was recognized (Bergaust et al., 2008). As the findings of Bergaust et al. (2008) indicated that gene expression played an important role in causing NO emission, it was concluded that the metabolic model should include genomic, proteomic and metabolic regulation. In this study, we have developed such a metabolic network model, which to our knowledge has not been attempted before for the denitrification pathway. The aims of the model were to test hypotheses on the most important factors in the regulation of the denitrification process during the transition from aerobic to anoxic conditions, to identify parameter values and to define a model framework that can be used to design further experiments.

## Methods

### Model development

The model was developed based on oxic/anoxic transition experiments with *A. tumefaciens* as described extensively in Bergaust et al. (2008). The experiments were performed in 120 ml serum flasks, containing growth medium supplemented with different concentrations of KNO_2_ or KNO_3_ and succinate as the only C-source. The initial oxygen concentration in the headspace was varied. Full experimental data-sets that were used for mathematical modeling were obtained from experiments with 1 and 7% oxygen in the headspace and 0.2, 1, and 2 mM of nitrite or nitrate. The experiments with no nitrite and nitrate and without initial oxygen were excluded because of less well-defined starting conditions (always some oxygen intrusion) and interference of presence of minimal nitrogen in the trace element solution. Inocula of fully dispersed aerobically grown cells were injected into the flasks. The headspace NO, N_2_O, O_2_, and CO_2_ concentrations were monitored during oxygen depletion and subsequent anoxic respiration. Measured NO concentrations in the nitrate experiments were corrected for calibration errors in the data that were originally presented by Bergaust et al. (2008). The calibration error led to an under-estimation of NO concentrations above 400 nM.

The experiment with 1% oxygen in the headspace (corresponding to ~10 μM oxygen in the liquid) and 1 mM nitrite as initial concentrations was used as reference experiment. The nitrite experiments were found more suitable for modeling because the concentrations of all nitrogen species involved were measured (NO, N_2_O) or could be calculated using mass balances (nitrite). In the nitrate experiments, nitrite concentrations and *nap* expression were unknown, complicating the interpretation of the experimental data. For the reference experiment gene expression of *NorB* and *NirK* was analysed using RT-PCR. Gene expression data of the experiment conducted at 1 mM nitrate and 1% initial oxygen concentration were used to identify *NirK* expression kinetics in relation to nitrite concentrations.

**Figure 1:**
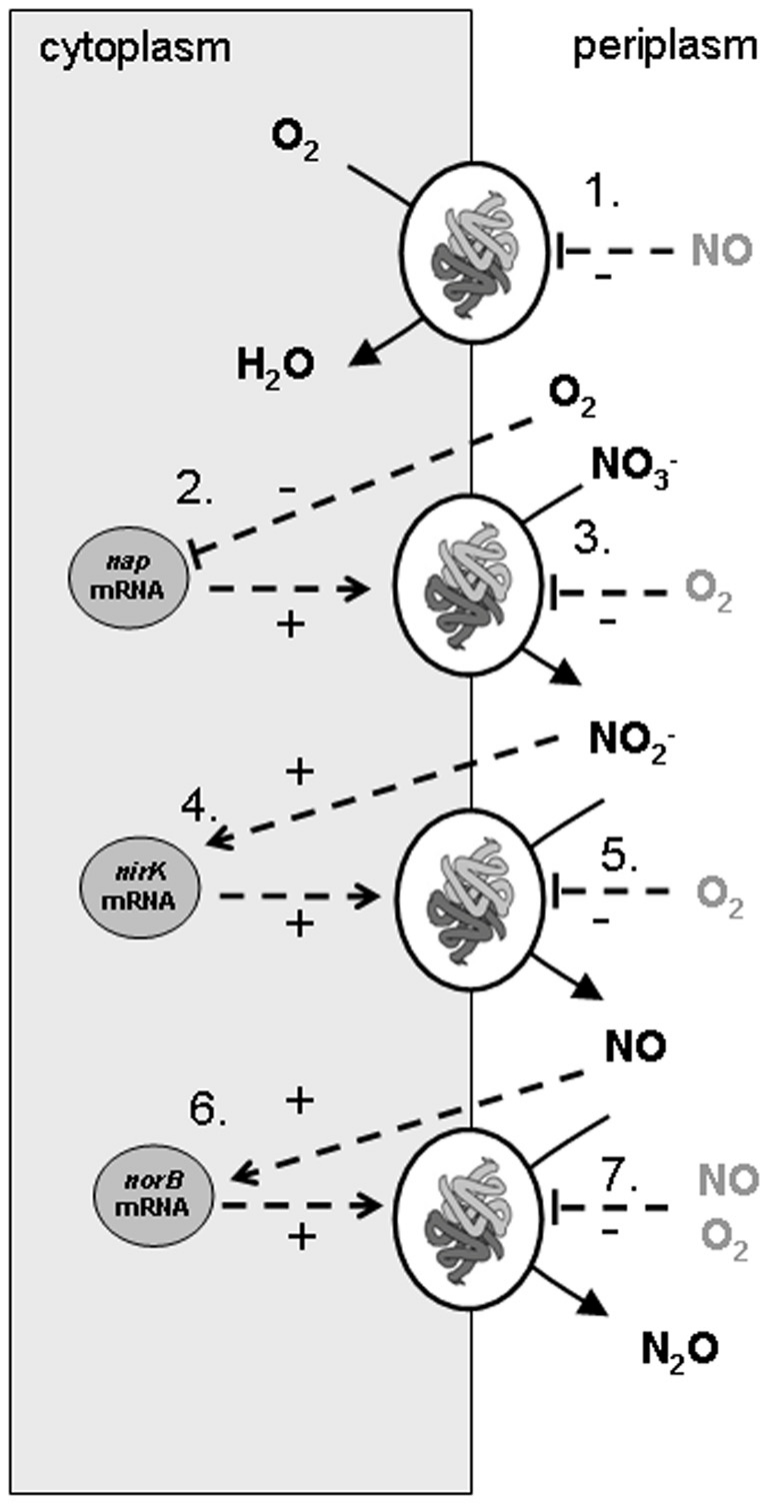
**Scheme of conversions and regulation of denitrification pathway in *Agrobacterium tumefaciens* as used in the metabolic model of the experiments**. The combination of experimental data and the metabolic model led to identification of *nirK* and *norB* transcription activated by their substrates, *nap* transcription by oxygen limitation, apparent (indirect, via electron transport chain) oxygen inhibition of the denitrification conversions and NO inhibition on the cytochrome c oxidase. The processes that were modeled are: (1) oxygen reduction, (2) nitrate reductase synthesis, (3) nitrate reduction, (4) nitrite reductase synthesis, (5) nitrite reduction, (6) NO reductase synthesis, (7) NO reduction.

The model developed considers that *A. tumefaciens* converts succinate (electron donor and carbon source) with oxygen (aerobic respiration) or with nitrate, nitrite, and NO (denitrification steps). In the experiments denitrification rates are changing over time due to availability of electron acceptors (O_2_, nitrate, nitrite, and NO) and varying enzyme concentrations. Consequently, denitrification is described in the metabolic model by two levels of cellular organization: (1) enzyme expression (in which DNA transcription and translation are lumped) and (2) enzyme activity leading to substrate conversion and microbial growth. The lumping of transcription and translation means that the model assumes that the rate of enzyme production is proportional to the mRNA level and that the response time for enzyme translation is insignificant. It is further assumed that the translation rate is equal for *nap*-, *nirK*-, and *norB*-transcripts. This simplification led to a good description of the reference experiment but could be responsible for the poor extrapolation of the model to other experiments. A scheme of the conversions and regulation factors of the denitrification pathway in *A. tumefaciens* as used in the metabolic model is shown in Figure 1.

Several biological and chemical reactions occur in the liquid according to the stoichiometry, rate expressions and parameters described in the next section. Because the model was developed gradually from simple to more complex, new factors affecting the process were only included if this resulted in an unequivocal improvement of the description of the experimental data. The goodness of fit was analyzed by calculating the sum of squared error (SSE) between the experimental data and the modeled values for the gaseous concentrations of O_2_, NO, N_2_O, and CO_2_. The model complexity was minimized to avoid the introduction of a large number of non-identifiable parameters.

### Model components

The model is based on batch (time-dependent) mass balances in the liquid and gas phase, for a number of chemical species, enzymes and microbial biomass. In the liquid phase the chemical species with concentrations changing in time are: oxygen (*C*_O_2__), nitrate (*C*_NO_3__), nitrite (*C*_No_2__), nitric oxide (*C*_NO_), nitrous oxide (*C*_N_2_O_), carbon dioxide (*C*_CO_2__) and bicarbonate (*C*_HCO_3__). All these concentrations are expressed in molar units. Time-dependent enzyme (nitrate reductase *E*_sat, nap_, nitrite reductase *E*_sat, nir_, NO reductase *E*_sat, nor_) and the *Agrobacterium* biomass concentrations (*C*_X_ in C-mol/L) are also considered. The model assumes a maximum enzyme concentration in the cell so that the relative dimensionless enzyme saturation, *E*_sat_, ranges from 0 (absence of enzyme) to 1. Succinate is present in excess and it is assumed not a rate limiting reactant, therefore it was not included in the kinetic model. It was also considered that the liquid is sufficiently buffered so that pH changes can be neglected and therefore H^+^ concentration *C_H_* can be considered constant in time (pH 7.5). In the gas phase there are four changing concentrations: oxygen (*c*_O_2__), nitric oxide (*c*_NO_), nitrous oxide (*c*_N_2_O_), and carbon dioxide (*c*_CO_2__).

### Model processes

#### Microbial conversions

The model considers the microbial conversion of succinate as electron donor and carbon source with oxygen (respiration), or with nitrate, nitrite and NO (denitrification steps) as electron acceptors.

The stoichiometric equations were derived from redox balances and theoretical knowledge on growth yields, by using the procedures described in Stouthamer (1979) and Heijnen (1999). The following theoretical values were applied: H^+^/ATP ratio = 4, ATP yield = 9.1 g dry weight biomass/mol ATP, proton translocation on oxygen 10 H^+^/NADH, 8 H^+^/FADH_2_, and during denitrification 6 H^+^/NADH and 4 H^+^/FADH_2_ (Wasser et al., 2002). It was assumed in the derivation of reaction stoichiometry that ammonium (present as trace element) was used for N incorporation into biomass. The biomass molar weight is 24.6 g dry weight/C-mol (CH_1.8_O_0.5_N_0.2_).

The reaction rates are based on conventional substrate affinity expressions (Monod or Michaelis-Menten) with linear dependency on the biomass concentration *C_X_* and the specific enzyme levels *E*_sat_. The electron acceptor consumption rates were assumed to be limited by the maximum succinate oxidation rate and therewith independent of the electron acceptor (Beun et al., 2000) as shown for *Paracoccus denitrificans* (Thomsen et al., 1994). The maximum specific succinate uptake rate was calibrated by fitting to the measured oxygen uptake profile.

For oxygen respiration, a competitive inhibition term for NO on oxygen respiration was applied. In the NO reduction process, substrate inhibition occurs at micromolar concentrations (Girsch and de Vries, 1997), and was modeled by standard Haldane kinetics. Because two NO molecules are consumed for generation of N_2_O, the reaction is second order with respect to NO (Girsch and de Vries, 1997). Oxygen inhibits nitrite and NO reduction on the conversion level, not enzyme synthesis level as it can be concluded based on RT-PCR for *NirK* and *NorB*. Due to the strong oxygen inhibition, a power function on the conventional inhibition terms was needed.

With these assumptions, the molar stoichiometry and the rates (mol succinate L^−1^ h^−1^) of the four considered microbial pathways are:

1. Aerobic conversion of succinate:
  $${\text{C}}_{4}{\text{H}}_{4}{\text{O}}_{4}^{2-}+1.2{\text{O}}_{2}+1.56{\text{H}}^{+}+0.44{\text{NH}}_{4}^{+}\to 2.2{\text{CH}}_{1.8}{\text{O}}_{0.5}{\text{N}}_{0.2}+1.8{\text{CO}}_{2}+\text{1}.{\text{68H}}_{2}\text{O}$$
  $$\text{with rate: }r_{\text{suc},{\text{ O}}_{2}}=q_{m}\times C_{X}\times \frac{{\text{C}}_{{\text{O}}_{2}}}{{\text{K}}_{{\text{O}}_{\text{2}}}\left(1+\frac{{\text{C}}_{\text{NO}}}{{\text{K}}_{\text{I},\text{ NO},{\text{ O}}_{\text{2}}}}\right)+{\text{C}}_{{\text{O}}_{2}}}$$
2. Nitrate reduction with succinate:
  $${\text{C}}_{4}{\text{H}}_{4}{\text{O}}_{4}^{2-}+\text{3}.{\text{23NO}}_{3}^{-}+1.64{\text{H}}^{+}+0.36{\text{NH}}_{4}^{+}\to \text{1}.{\text{8CH}}_{\text{1}.\text{8}}{\text{O}}_{\text{0}.\text{5}}{\text{N}}_{\text{0}.\text{2}}+\text{3}.{\text{23NO}}_{2}^{-}+\text{2}.{\text{2CO}}_{2}+1.92{\text{H}}_{2}\text{O}$$
  with rate:
  $$r_{\text{suc},\text{ NAP}}=q_{m}\times C_{X}\times E_{\text{sat},\text{ NAP}}\times \frac{{\text{C}}_{{\text{NO}}_{3}}}{{\text{K}}_{{\text{NO}}_{3}}+{\text{C}}_{{\text{NO}}_{3}}}\times \frac{{\text{K}}_{\text{I},{\text{ O}}_{2},\text{ NAP}}^{\text{nNAP}}}{{\text{K}}_{\text{I},{\text{ O}}_{2},\text{ NAP}}^{\text{nNAP}}+{\text{C}}_{{\text{O}}_{2}}^{\text{nNAP}}}$$
3. Nitrite reduction with succinate:
  $${\text{C}}_{4}{\text{H}}_{4}{\text{O}}_{4}^{2-}+6.45{\text{NO}}_{2}^{-}\text{+ 8}.{\text{09H}}^{+}\text{+ 0}.{\text{36NH}}_{4}^{+}\to \text{1}.{\text{8CH}}_{\text{1}.\text{8}}{\text{O}}_{\text{0}.\text{5}}{\text{N}}_{0.2}+6.45\text{NO}+2.2{\text{CO}}_{2}+5.15{\text{H}}_{2}\text{O}$$
  with rate:
  $$r_{\text{suc},\text{ NIR}}=q_{m}\times C_{X}\times E_{\text{sat},\text{ NIR}}\times \frac{{\text{C}}_{{\text{NO}}_{2}}}{{\text{K}}_{{\text{NO}}_{2}}+{\text{C}}_{{\text{NO}}_{2}}}\times \frac{{\text{K}}_{\text{I},{\text{ O}}_{2},\text{ NIR}}^{\text{nNIR}}}{{\text{K}}_{\text{I},{\text{ O}}_{2},\text{ NIR}}^{\text{nNIR}}+{\text{C}}_{{\text{O}}_{2}}^{\text{nNIR}}}$$
4. NO reduction with succinate:
  $${\text{C}}_{4}{\text{H}}_{4}{\text{O}}_{4}^{2-}+6.45\text{NO}+1.64{\text{H}}^{+}+0.36{\text{NH}}_{4}^{+}\to 1.8{\text{CH}}_{1.8}{\text{O}}_{0.5}{\text{N}}_{0.2}+3.23{\text{N}}_{2}\text{O}+2.2{\text{CO}}_{2}+1.92{\text{H}}_{2}\text{O}$$
  with rate:
  $$r_{\text{suc},\text{ NOR}}=q_{m}\times C_{X}\times E_{\text{sat},\text{ NOR}}\times \frac{{\text{C}}_{\text{NO}}^{2}}{{\left[{\text{C}}_{\text{NO}}\times \left(1+\frac{{\text{C}}_{\text{NO}}}{{\text{K}}_{\text{I},\text{ NO}}}\right)+{\text{K}}_{\text{NO}}\right]}^{2}}\times \frac{{\text{K}}_{\text{I},{\text{ O}}_{2},\text{ NOR}}}{{\text{K}}_{\text{I},{\text{ O}}_{2},\text{ NOR}}+{\text{C}}_{{\text{O}}_{2}}}$$

#### Enzyme synthesis

For the enzyme synthesis Michaelis–Menten kinetics was assumed as a function of the enzyme inducer concentrations. A further saturation factor (1-*E*_sat_) was introduced to limit the enzyme concentration in the cells (Wild et al., 1994). The model assumes a dimensionless enzyme saturation, in which the enzyme concentration ranges from absence of enzyme (*E*_sat_ = 0) to maximum enzyme concentration in the cell (*E*_sat_ = 1). The experimentally observed rates of expression were different for the individual denitrification enzymes, i.e., *nor* expression is quicker than *nir* expression. The *NAP*, *NirK*, and *NorB* transcription were activated by their respective substrates nitrate, nitrite, and NO. Additionally the *nap* transcription was inhibited by oxygen. Because oxygen respiration is constitutively expressed, enzyme synthesis is not considered for this process. The model does not take enzyme decay into account because this was not necessary to describe the experimental data. Additionally, experimental data with repetitive oxygen addition to batch flasks indicated that enzyme decay for oxygen respiration is negligible for the time-scale of these experiments.

With these assumptions, the enzyme synthesis rates (h^−1^) are:

1. Synthesis of nitrate reductase *nap*:
  $$\frac{dE_{\text{sat},\text{ NAP}}}{dt}=v_{\text{m},\text{ NAP}}\times \frac{{\text{C}}_{{\text{NO}}_{3}}}{{\text{K}}_{{\text{NO}}_{3},\text{ NAP}}+{\text{C}}_{{\text{NO}}_{3}}}\times \frac{{\text{K}}_{\text{I},{\text{ O}}_{2},\text{ NAP}}}{{\text{K}}_{\text{I},{\text{ O}}_{2},\text{ NAP}}+{\text{C}}_{{\text{O}}_{2}}}\times (1-E_{\text{sat},\text{ NAP}})$$
2. Synthesis of nitrite reductase *nir*:
  $$\frac{dE_{\text{sat},\text{ NIR}}}{dt}=v_{\text{m},\text{ NIR}}\times \frac{{\text{C}}_{{\text{NO}}_{2}}}{{\text{K}}_{{\text{NO}}_{2},\text{ NIR}}+{\text{C}}_{{\text{NO}}_{2}}}\times (1-E_{\text{sat},\text{ NIR}})$$
3. Synthesis of NO reductase *nor*:
  $$\frac{dE_{\text{sat},\text{ NOR}}}{dt}=v_{\text{m},\text{ NOR}}\times \frac{{\text{C}}_{\text{NO}}}{{\text{K}}_{\text{NO},\text{ NOR}}+{\text{C}}_{\text{NO}}}\times (1-E_{\text{sat},\text{ NOR}})$$

#### NirK transcription

**Figure 2:**
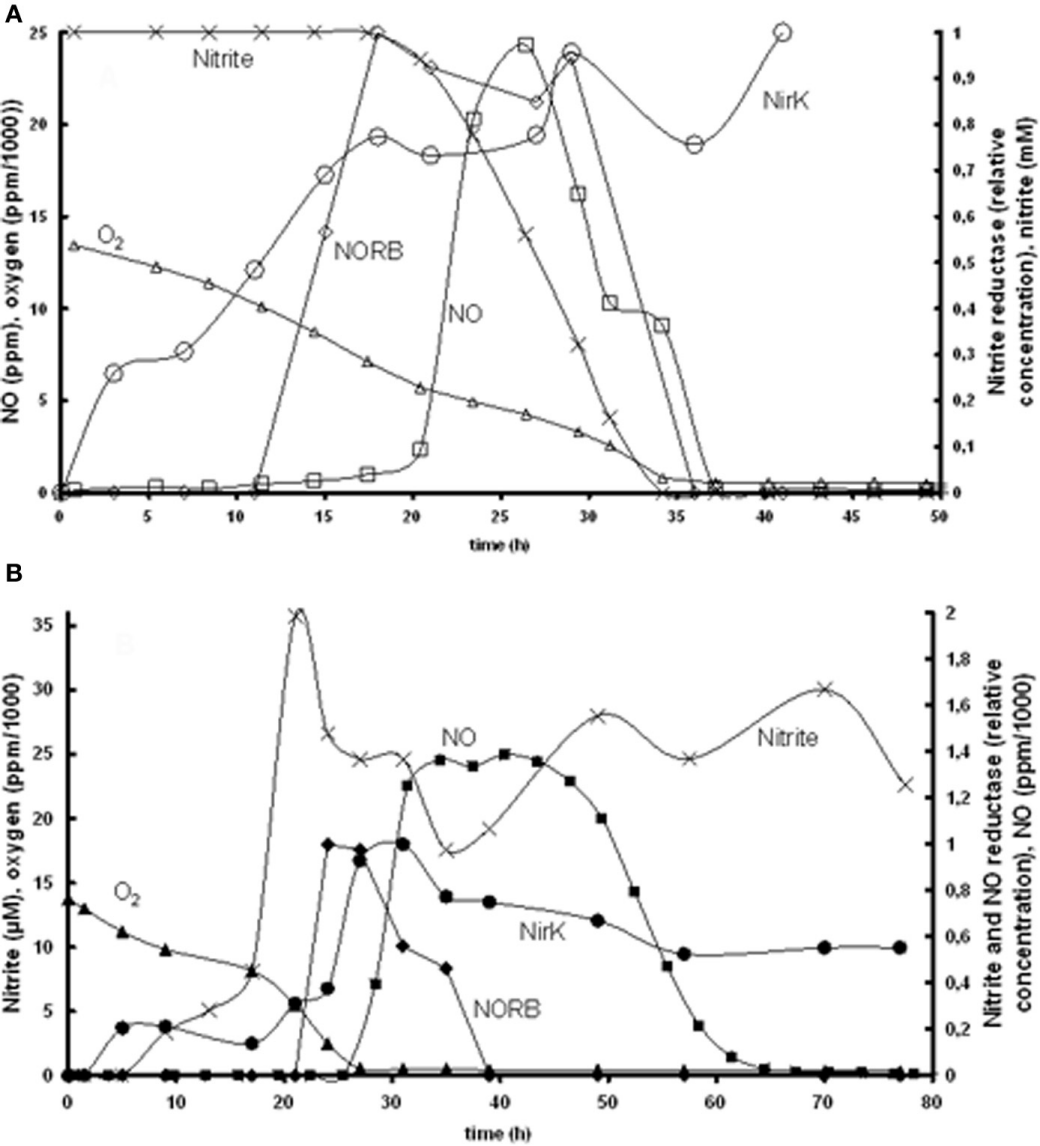
**Measured *nirK* and *norB* transcription together with concentrations of nitrate, nitrite, N_2_O, and O_2_ in time. (A)** Experiment with 1% gas phase oxygen and 1 mM nitrite: nitrite (×), NirK (○), NORB (◊) NO (□), oxygen (∆). **(B)** Experiment with 1% gas phase oxygen and 1 mM nitrate; nitrite (×), NirK (•), NORB (♦), NO (■), oxygen (▲). Due to required amount of biomass for PCR analysis these experiments comprised several parallel flasks, see Bergaust et al. (2008) for details about the experiments.

Based on the experimental data of Bergaust et al. (2008) it was hypothesized that the transcription of *nirK* is activated by its substrate nitrite. This could be identified based on the experiments at 1% initial oxygen in the gas phase combined with 1 mM nitrite or nitrate (Figure 2). The substrate concentration dependency of *nirK* transcription was modeled using saturation kinetics. In the nitrite experiment, *nirK* is rapidly transcribed from the start. The maximum specific *nirK* transcription rate was identified by the increase in *nirK* mRNA at non-limiting nitrite concentration (first 20 h in Figure 2). In the nitrate experiment *nirK* is only transcribed when the nitrite concentration has increased after nitrate reduction is initiated. The affinity constant for nitrite of *nirK* transcription (see Table 1) was deduced from the data obtained from the nitrate experiment (Figure 2, panel **B**). Here it can be seen that *nirK* transcription increases upon the increase in nitrite concentration.

**Table 1:** **Model parameters**.

**Definition**	**Symbol**	**Value**	**Unit**
**MICROBIAL CONVERSIONS**			
Maximum specific succinate utilization rate	*q*_*m*_	0.066	(mol succ.)(C-mol biomass)^−1^h^−1^
Oxygen respiration			
Monod saturation coefficient for oxygen	*K*_O_2__	8.28	μM
Inhibition coefficient of O_2_ respiration by NO	*K*_I,NO,O_2__	0.0174	μM
Nitrate reduction			
Monod saturation coefficient for nitrate	*K*_NO_3__	13000	μM
Inhibition coefficient by O_2_	*K*_I,O_2_,NAP_	4	μM
Exponent for oxygen inhibition	*nNAP*	4	
Nitrite reduction			
Monod saturation coefficient for nitrite	*K*_NO_2__	880	μM
Inhibition coefficient by O_2_	*K*_I,O_2_,NIR_	3.58	μM
Exponent for oxygen inhibition	*nNIR*	3.7	-
Nitric oxide reduction			
Monod saturation coefficient for NO	*K*_NO_	0.0081	μM
Inhibition coefficient by O_2_	*K*_I,O_2_,NOR_	1.0	μM
Inhibition coefficient by NO	*K*_I,NO_	20	μM
**ENZYME SYNTHESIS**			
Nitrate reductase			
Maximum enzyme synthesis rate	*v*_m,NAP_	1/15	h^−1^
Saturation coefficient for nitrate	*K*_NO_3_,NAP_	0.00001	μM
Inhibition coefficient by O_2_	*K*_I,O_2_,NAP_	1.0	μM
Nitrite reductase			
Maximum enzyme synthesis rate	*v*_m,NIR_	1/15	h^−1^
Saturation coefficient for nitrite	*K*_NO_2_,NIR_	50	μM
Nitric oxide reductase			
Maximum enzyme synthesis rate	*v*_m,NOR_	1	h^−1^
Saturation coefficient for nitric oxide	*K*_NO,NOR_	0.054	μM
Saturation coefficient for NOR enzyme synthesis (only for nitrate experiments)	*K*_O_2_,NOR_	400	μM
**CHEMICAL CONVERSIONS (Parkhurst and Appelo, 1999)**			
Rate coefficient for CO_2_ hydration	*k*_car_	10^14^	h^−1^
Equilibrium constant CO_2_ / HCO_3_^−^	*K*_a,car_	10^−6.36^	M
**EXPERIMENTAL PARAMETERS**			
Initial concentrations			
Oxygen in gas	*c*_O_2_,0_	1 and 7	% in gas phase
CO_2_ in gas	*c*_CO_2_,0_	0	% in gas phase
Nitric and nitrous oxides in gas	*c*_NO,0_, *c*_N_2_O,0_	0	% in gas phase
O_2_, NO, N_2_O and CO_2_ in liquid	*C*_O2,0_, *C*_NO,0_, *C*_N_2_O,0_, *C*_CO_2_,0_	in equilibrium with gas phase	
Nitrate	*C*_NO_3_,0_	0.2, 1 and 2	mM
Nitrite	*C*_NO_2_,0_	0.2, 1 and 2	mM
Biomass	*C*_*X*,0_	0.25	mC-mol L^−1^
Enzymes	*E*_sat,NAP,0_, *E*_sat,NIR,0_, *E*_sat,NOR,0_	0	–
Reactor geometry	–	–	–
Liquid volume	*V*_*L*_	0.05	L
Gas volume	*V*_*G*_	0.07	L
**PHYSICAL PARAMETERS (Sander, 1999)**			
Mass transfer coefficient	*k*_*L*_*a*	19.8	h^−1^
Henry coefficient of N_2_O	*H*_N_2_O_	1.74	M_*gas*_ M^−1^_*aq*_
Henry coefficient of NO	*H*_NO_	21	M_*gas*_ M^−1^_*aq*_
Henry coefficient of O_2_	*H*_O_2__	33	M_*gas*_ M^−1^_*aq*_
Henry coefficient of CO2	*H*_CO_2__	1.2	M_*gas*_ M^−1^_*aq*_

Our observation that nitrite activates *nirK* transcription in *A. tumefaciens* does not correlate with the observations of Baek and Shapleigh (2005). These authors suggested that NO induces *nir* expression as well as *nor* expression (see next paragraph). However, it is known that the two genes are differentially regulated (Baek et al., 2008) and our experimental data clearly show *nir* expression before NO appearance. Also a two-step mechanism can be proposed with low level *nirK* transcription when nitrite is present and increased transcription when NO increases due to nitrite reduction (as postulated for *Rhodobacter sphaeroides*, Baker et al., 1998). The *nirK* transcription measurements do show a low transcription level during the first hours when oxygen is still present (Figure 2) which can support this hypothesis. It cannot be excluded based on the experimental data that decrease in oxygen concentration also plays a role in the transcription regulation.

#### NorB transcription

**Figure 3:**
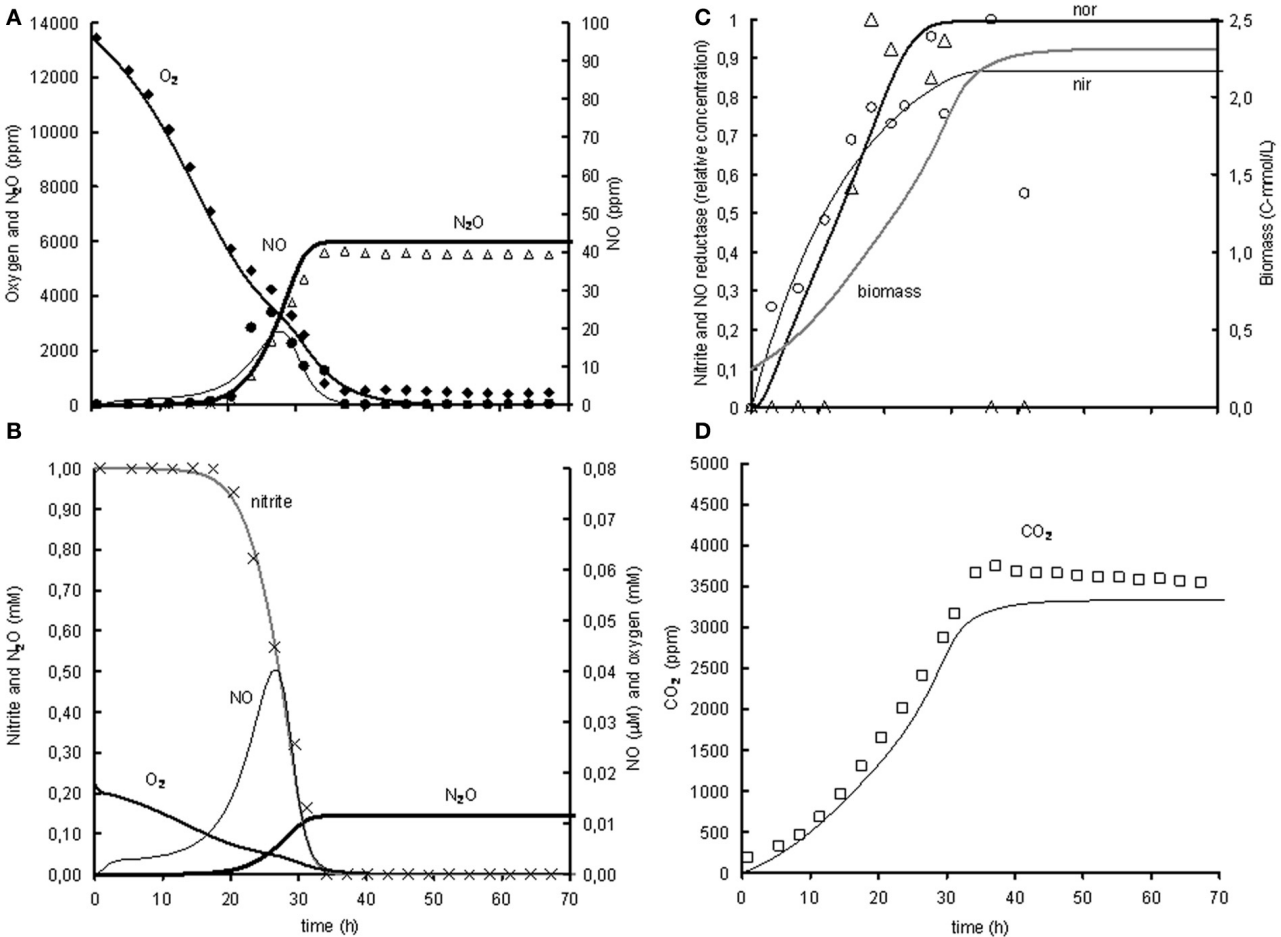
**Modeled (lines) and measured (points) concentrations during experiment with 1% gas phase oxygen and 1mM nitrite**. **(A)** Gas phase concentrations of O_2_ (♦), NO (•), and N_2_O (Δ). **(B)** Liquid concentrations of N_2_O (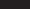), O_2_ (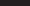), nitrite (×, 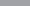), and NO (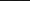). **(C)** Liquid concentrations of expressed *nir* (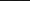), measured *nirK* mRNA (○), expressed *nor* (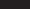), measured *norB* mRNA (ρ), and biomass (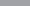). **(D)** Gas phase concentration of CO_2_ (□). The fit between the modeled data and the experimental data was assessed using the *R*^2^-value: R^2^ O_2_ = 0.99, R^2^ NO = 0.80, R^2^ N_2_O = 0.93, R^2^ CO_2_ = 0.97 with $R^{\text{2}}=1-\frac{\sum {\left({\text{C}}_{\exp}-{\text{C}}_{\text{model}}\right)}^{2}}{\sum {\left({\text{C}}_{\exp}-{\bar{\text{C}}}_{\text{model}}\right)}^{2}}$.

The induction of NO reductase expression by NO in *A. tumefaciens*, was previously shown (Baek and Shapleigh, 2005). The activation of *norB* transcription by NO can be deduced from panel A2 in Figure 2, where *norB* transcripts appear as soon as NO is measured. However, NO concentrations are very low, meaning that the affinity for NO is very high. These observations suggest that nitrite activates *nirK* transcription and NO stimulates *norB* transcription, which leads to a satisfactory model fit for the reference experiment (Figure 3). However, as indicated before, the enzyme synthesis parameters could not accurately be identified due to limited experimental data during the transition period. The maximum rate of transcription of *norB* is higher than the transcription rate for *nirK.* This can clearly be seen from the rapid increase in *norB* concentration after NO is detected, while *nirK* increases slower despite continuous nitrite presence.

#### Chemical conversions

The aqueous equilibrium between CO_2_ and HCO^−^_3_ was introduced in order to calculate the concentration of produced CO_2_ in the gas phase as a function of pH:

$${\text{CO}}_{2}+{\text{H}}_{2}\text{O}⇌{\text{HCO}}_{3}^{-}+{\text{H}}^{+}$$

$$\text{with rate: }r_{\text{car}}=k_{\text{car}}\times \left({\text{C}}_{{\text{CO}}_{2}}-\frac{{\text{C}}_{{\text{HCO}}_{3}}{\text{C}}_{H}}{K_{\text{a},\text{ car}}}\right)$$

Because compared with the time scale of the whole process this equilibrium is very fast, an arbitrarily very large value was set for *k*_car_.

### Mass balances for chemical and microbial components

In the liquid volume (batch operation) the following mass balances are solved to find the concentrations of chemical species and biomass:

$$\begin{array}{l} \;\frac{d{\text{C}}_{{\text{O}}_{2}}}{dt}=-1.2r_{\text{suc},{\text{ O}}_{2}}+r_{\text{tr},{\text{ O}}_{2}}\\ \;\frac{d{\text{C}}_{{\text{NO}}_{3}}}{dt}=-3.23r_{\text{suc},\text{ NAP}}\\ \;\frac{d{\text{C}}_{{\text{NO}}_{2}}}{dt}=3.23r_{\text{suc},\text{ NAP}}-6.45r_{\text{suc},\text{ NIR}}\\ \;\frac{d{\text{C}}_{\text{NO}}}{dt}=6.45r_{\text{suc},\text{ NIR}}-6.45r_{\text{suc},\text{ NOR}}+r_{\text{tr},\text{ NO}}\\ \;\frac{d{\text{C}}_{{\text{N}}_{2}\text{O}}}{dt}=3.23r_{\text{suc},\text{ NOR}}+r_{\text{tr},{\text{ N}}_{\text{2}}\text{O}}\\ \;\frac{d{\text{C}}_{{\text{CO}}_{2}}}{dt}=1.8r_{\text{suc},{\text{ O}}_{\text{2}}}+2.2\left(r_{\text{suc},\text{ NAP}}+r_{\text{suc},\text{ NIR}}+r_{\text{suc},\text{ NOR}}\right)-r_{\text{car}}+r_{\text{tr},{\text{ CO}}_{2}}\\ \frac{d{\text{C}}_{{\text{HCO}}_{3}}}{dt}=r_{\text{car}}\\ \;\frac{d{\text{C}}_{X}}{dt}=2.2r_{\text{suc},{\text{ O}}_{2}}+1.8\left(r_{\text{suc},\text{ NAP}}+r_{\text{suc},\text{ NIR}}+r_{\text{suc},\text{ NOR}}\right) \end{array}$$

For the gas volume (batch operation) there is only mass exchange with the liquid phase, for each gaseous species (*i* = O_2_, CO_2_, NO, N_2_O):

$$\frac{dc_{i}}{dt}=-r_{\text{tr},\;i}\frac{V_{L}}{V_{G}}$$

The gas-liquid mass transfer rate is:

$$r_{\text{tr},\;i}=k_{L}a\left(\frac{c_{i}}{H_{i}}-C_{i}\right)$$

The model solution and parameter estimation procedure were implemented numerically in Matlab (The Mathworks, Inc., Natick, MA, USA). The system of ordinary differential equations is solved from the initial conditions (*C*_*i*_,_0_, *c*_*i*_,_0_, and *E*_*sat*,*i*,0_), including all reactions and mass transfer terms with the kinetic, physical and operational parameters presented in Table 1.

### Model parameters

The rate parameters for microbial and enzyme kinetics were estimated based on the experimental data using the multivariable constrained optimization routine based on sequential quadratic methods (function fmincon from Matlab). The experimental parameters were based on the actual experiments. The model code can be found in Appendix I.

## Results

### Metabolic model of the base experiment

The model was based on the experiment with 1% oxygen in the headspace and 1 mM nitrite as initial concentrations (see Figure 3). The goodness of fit was analysed by calculating the SSE between the experimental data and the modeled values for the gaseous concentrations of O_2_, NO, N_2_O, and CO_2_. The gaseous concentrations of O_2_, CO_2_, and N_2_O are described very well by the metabolic model (*R*^2^ > 0.93), while the best fit for description of the NO concentration in the head-space was an *R*^2^ of 0.8 (see heading of Figure 3 for goodness-of-fit data). As the NO accumulation is extremely small compared to the overall flux through the NO pool and several processes are dependent on the NO concentration, it was difficult to capture the trend of NO accumulation (for example, the NO accumulation between 23 and 26 h was 0.2% of the overall flux through the NO pool which is derived from the N_2_O accumulation). Also the modeled nitrite concentration and *NorB* and *nirK* transcription fit well with the measured data (see Figures 3B and C). The biomass growth during the experiment (see Figure 3C) clearly affects the conversion rates, which for example can be seen from the increase in volumetric oxygen uptake rates during the experiment.

A sensitivity analysis was performed to analyse the identifiability of the different parameters. For this purpose the normalized sensitivity was calculated. In the normalization the SSE is corrected for the average of the concentration of the different species to prevent bias due to difference in absolute concentration. The percentual change of the SSE_total upon a 10% change of the separate parameters is presented in Table 2. This normalized change is an indication of the sensitivity and identifiability of the parameter.

$$\text{SSE}\_\text{total}=\sum \text{​}(\frac{{\left({\text{C}}_{e\text{xp},{\text{ O}}_{2}}-{\text{C}}_{\text{model},{\text{ O}}_{2}}\right)}^{2}}{\bar{{\text{C}}_{\text{exp},{\text{ O}}_{2}}}}+\frac{{\left({\text{C}}_{\exp,{\text{ N}}_{2}\text{O}}-{\text{C}}_{\text{model},{\text{ N}}_{2}\text{O}}\right)}^{2}}{\bar{{\text{C}}_{\text{exp},{\text{ N}}_{2}\text{O}}}}+\frac{{\left({\text{C}}_{\exp,\text{ NO}}-{\text{C}}_{\text{model},\text{ NO}}\right)}^{2}}{\bar{{\text{C}}_{\text{exp},\text{ NO}}}}+\frac{{\left({\text{C}}_{\exp,{\text{ CO}}_{2}}-{\text{C}}_{\text{model},{\text{ CO}}_{2}}\right)}^{2}}{\bar{{\text{C}}_{\text{exp},{\text{ CO}}_{2}}}})\text{normalized}\;\text{change}=\frac{\left({\text{SSE}}_{10\%\;\text{change}}-{\text{SSE}}_{\text{fit}}\right)}{{\text{SSE}}_{\text{fit}}}$$

**Table 2:** **Normalized change (%) in sum of squared errors for the parameters in the metabolic model**.

**Parameter change**	**Change SSE total (%)**		**Change SSE oxygen (%)**		**Change SSE NO (%)**		**Change SSE N_2_O (%)**		**Change SSE CO_2_ (%)**	
	**−10%**	**+10%**	**−10 %**	**+10%**	**−10%**	**+10%**	**−10%**	**+10%**	**−10%**	**+10%**
k_nir	0.2	0.0	1.7	−1.0	4.5	−3.0	−0.8	0.7	−0.5	0.5
k_nor	0.4	0.1	−0.2	0.7	−0.1	0.4	−1.7	2.0	3.2	−2.6
q_succ	139	88	180	148	115	22	135	155	105	−40
K_NO	0.7	1.7	1.8	1.7	4.4	2.2	6.1	−3.2	−6.3	6.6
K_NO_2_	0.5	−0.0	−1	2	−3	6	2	−2	0.4	−0.2
K_NO_2__NIR	0.00	0.00	−0.06	0.06	−0.17	0.18	0.05	−0.05	0.02	−0.02
K_NO_NorB	0.0	0.3	0.7	−0.2	0.0	0.2	1.9	−1.4	−2.5	2.5
K_O_2_	20	26	42	32	−22	41	45	9	−26	35
KI_NO	0.00	0.00	0.00	0.00	−0.03	0.03	−0.02	0.02	0.02	−0.02
KI_NO_resp	4	1	3	3	14	−11	−3	8	10	−8
KI_O_2__NIR	5	2	16	1.	32	−20	1	2	−3	4.6
KI_O_2__NOR	4	2	5	3	8	15	−4	9	11	−8
n_O_2__nir	0.3	1.3	−0.7	4.5	3.2	−1.2	−1.5	1.9	3.0	−2.2

This analysis demonstrated that the SSE increased dramatically upon changing the values of maximum biomass specific substrate uptake rate and the affinity constant for oxygen of the terminal oxidase. These parameters could be accurately identified since their values determine the exact time at which denitrification starts. Also the inhibition constant for NO on the oxygen respiration and the (apparent) inhibition of oxygen on nitrite and NO reduction were well identifiable. The substrate inhibition constant for NO reductase was poorly identifiable from this experiment (i.e., no significant change in the SSE when the parameter value was changed) because the NO concentrations do not reach the inhibitive concentration.

### Application of the model to other experiments

**Figure 4:**
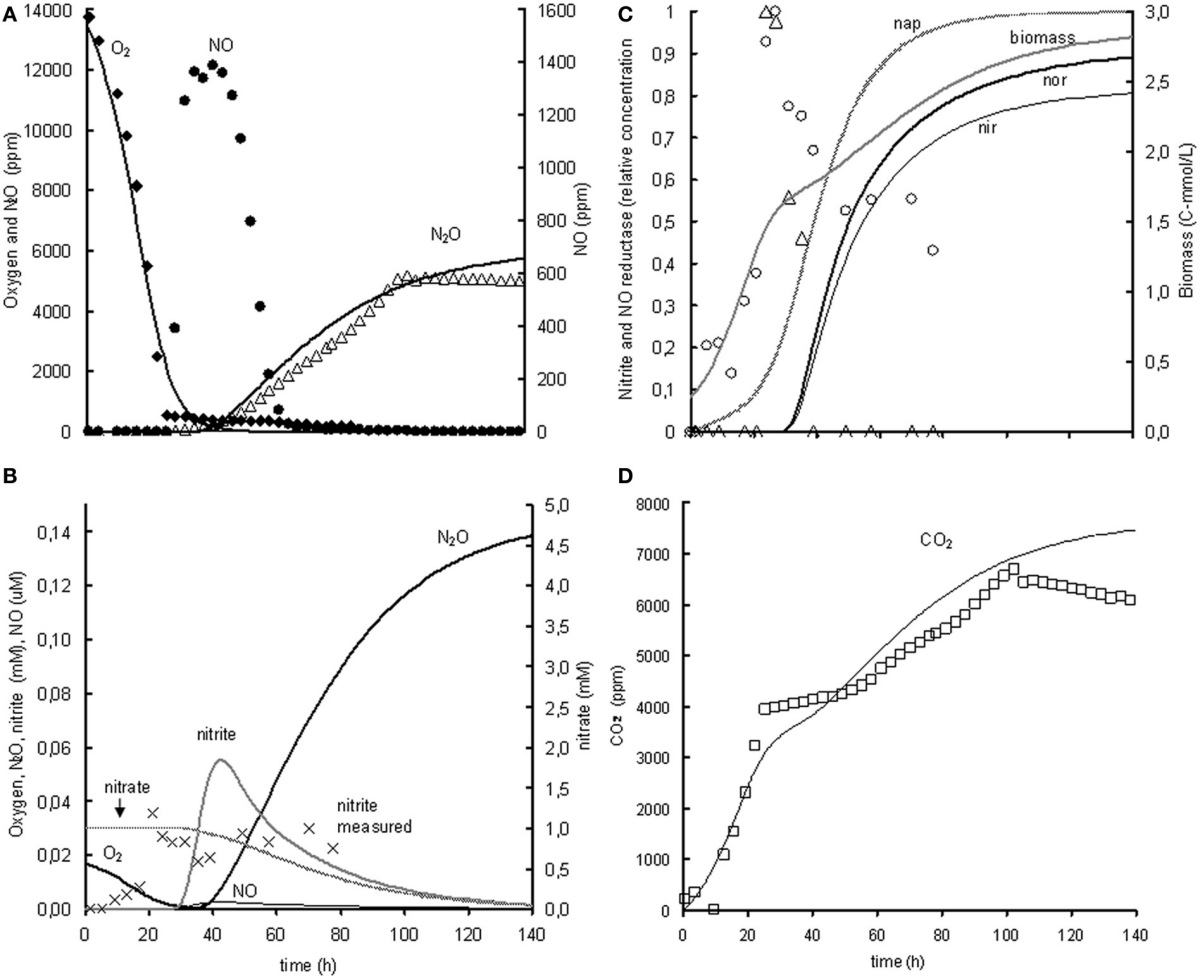
**Modeled (lines) and measured (points) concentrations when extrapolating the metabolic model to the experiment with 1% gas phase oxygen and 1 mM nitrate**. **(A)** Gas phase concentrations of O_2_ (♦), NO (•), and N_2_O (Δ). **(B)** Liquid concentrations of N_2_O (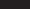), O_2_ (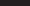), nitrate (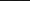), nitrite (×, 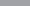), and NO (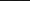). **(C)** Liquid concentrations of expressed *nap* (– –), expressed *nir* (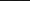), measured *nirK* mRNA (○), expressed *nor* (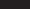), measured *norB* mRNA (Δ), and biomass (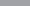). **(D)** Gas phase concentration of CO_2_ (□). The fit between the modeled data and the experimental data was assessed using the *R*^2^-value: *R*^2^ O_2_ = 0.98, *R*^2^ NO = –0.29, *R*^2^ N_2_O = 0.85, *R*^2^ CO_2_ = 0.80 with $R^{2}=1-\frac{\sum {\left(C_{\exp}-{\text{C}}_{\text{model}}\right)}^{2}}{\sum {\left(C_{\exp}-{\bar{\text{C}}}_{\text{model}}\right)}^{2}}$. The negative *R*^2^ for NO is caused by the poor description of the experimental NO concentrations by the model. Consequently, the sum of squared errors for the model description was larger than the sum of the variance of the experimental values.

The metabolic model is based on the experiment with 1% oxygen gas phase and 1 mM nitrite as initial concentrations. Subsequently, the model was extrapolated to the experiment with 1% oxygen gas phase and 1 mM nitrate as initial concentrations. When modeling the nitrate experiment, the parameters identified in the nitrite experiment were used and only new parameters were added for the reduction of nitrate to nitrite. The modeled behavior of the oxygen uptake and N_2_O and CO_2_ production showed a relatively good correlation with the experimental data but the modeled NO concentrations were much lower than the measured NO concentrations (see Figure 4). This is further discussed in the model limitations section (“Model Limitations and Outlook”).

The model was also applied to experiments at 0.2 and 2 mM initial nitrite and nitrate and 1 and 7% of initial oxygen gas phase. The complete comparison between model results and experimental data on this additional data-set is shown in Appendix II. The model could describe oxygen consumption in these experiments relatively well, but the predicted NO and N_2_O emission did not correlate well with the experimental data. Obviously, a better fit could be generated if parameter values were adjusted for these individual experiments (now the parameter values were fitted on the 1% oxygen and 1 mM nitrite/nitrate experiments). However, this would only generate limited additional insight. Especially the parameters involved in the enzyme synthesis could not be accurately defined (see Table 2) due to few experimental data. This might cause the poor fit of the NO and N_2_O concentrations when the model was used for different initial conditions, as enzyme synthesis in the model was highly dependent on the oxygen, nitrite, and NO concentrations. Additionally, in the experiments with higher initial oxygen concentrations oxygen depletion proceeded faster due to increased biomass presence, potentially leading to a different metabolic state of the cell (see “Model Limitations and Outlook” for further explanation).

These observations do indicate that some additional phenomena should be included in the model, to have a broader application range of the model. To identify additional phenomena additional experiments are needed, as described in the model limitation section (“Model Limitations and Outlook”).

## Discussion

The use of the metabolic model and rate-based analysis of the experimental data enabled quantitative insight of the processes and increased understanding of the interdependence of the processes occurring during transition. For example, the NO accumulation was very tightly dependent on the combination of respiration and denitrification rates, as it also plays an inhibitory role on respiration. The model was used to test hypotheses that were based on the experimental observations (Bergaust et al., 2008) on the regulation of several phenomena in enzyme transcription and enzyme conversion kinetics, which are presented in the next section.

### Enzyme transcription kinetics and activators

#### Absence of interdependence of regulation of nitrite and NO reduction

Generally it is assumed that nitrite and NO reduction are controlled interdependently, both at the expression and the enzyme activity level. Interdependent regulation of both enzymes is thought to secure minimal NO accumulation (Zumft, 1997). In *P. denitrificans* for example, nitrite reduction and NO reduction are interdependent; *nor* deprived mutants also stop nitrite reduction preventing accumulation of toxic levels of NO (de Boer et al., 1996). In *A. tumefaciens* such a mechanism seems to be absent since NO clearly accumulates to toxic levels upon rapid oxygen depletion and in the presence of elevated nitrite concentrations (see Appendix II).

The expression of nitrate reductase (periplasmic dissimilatory nitrate reductase, *nap*) is in many organisms regulated by nitrate and oxygen limitation (Zumft, 1997). Oxygen limitation as an inducer of *nap* transcription led to a slightly better fit of the modeled values to the experimental data than when only nitrate-induced *nap* transcription. Because nitrate was always present in the experiments with nitrate reduction and no *nap* transcription measurements were performed, the effect of nitrate could not be identified and subsequently only oxygen limitation was used in the metabolic model for description of *nap* transcription.

### Enzyme kinetics

#### NO inhibits oxygen respiration

NO clearly inhibits oxygen reduction as shown in the experiments with 1% oxygen atmosphere and 1 mM nitrite (Figure 3) and 2 mM nitrite (see Appendix II). NO is an important signaling compound and has been shown to inhibit terminal oxidases in mitochondria of eukaryotes (Giuffre et al., 1996; Sarti et al., 2000) and bacterial terminal oxidases (Borisov et al., 2004). Including NO inhibition in the metabolic model greatly improved the description of the experimental data. At the lower nitrite concentrations, *nor* expression is adequately fast to prevent NO accumulation up to inhibitory levels. In the nitrate experiments NO inhibition on oxygen respiration could not be identified because NO is only formed in significant amounts after oxygen is depleted.

#### NO inhibition of nitrate reductase does not occur

In the paper of Bergaust et al. (2008) inhibition of nitrate reductase by NO was hypothesized as an explanation why NO did not further accumulate anymore but stayed at a constant concentration. The model runs clearly showed that this cannot be the case, as even though the concentration was constant there was a large flux through the NO-pool. In other words, the production and consumption of NO was constant, but nitrate was still consumed and N_2_O produced.

#### Oxygen inhibits all denitrification conversions

*NirK* and *norB* are already transcribed in the presence of oxygen (see previous section). Nevertheless, denitrification activity mainly occurs when oxygen concentrations are low. This suggests that oxygen apparently inhibits the denitrification reactions. However, literature information indicates that oxygen does not directly inhibit the enzymes in the denitrification pathways, except for N_2_O reductase (Zumft, 1997). Consequently, the oxygen inhibition observed is likely the result of preferred electron flow towards aerobic respiration rather than anoxic respiration.

#### NO accumulates after rapid oxygen depletion

Exceptionally high NO accumulation occurred in the experiments with a higher initial oxygen concentration (7%, see Appendix II). These experiments are characterized by very rapid oxygen depletion, because of the high biomass accumulation as a result of the long oxic growth phase. Apparently, the cell metabolism cannot respond adequately to such a rapid oxygen depletion leading to imbalanced expression of the denitrification pathway. It seems that *norB* transcription and translation suffers most heavily from this imbalance, possibly because NO reduction is the last conversion in the denitrification pathway and the *norB* transcription is activated by NO.

In the 1 mM nitrate experiment with 1% initial oxygen in the gas phase (Figure 4, panel **C**), it appears that *norB* transcription even stops when oxygen is depleted, even though NO and nitrite are still present.

Experiments with repeated oxygen addition indicated that oxygen addition was beneficial for recovery of the denitrification metabolism after cells had suffered from rapid oxygen depletion (see Appendix III). Oxygen addition led to an increase of the NO reduction rates. This should be further investigated in experiments where quantification of *nor* expression is included.

A beneficial role of residual oxygen presence during transition from aerobic to anoxic respiration on nitrate in *A. tumefaciens* was also observed by Baek and Shapleigh (2005). In various *Pseudomonas* species it was observed that expression of denitrification enzymes during oxygen limitation can lead to low activity of *nor*. In these experiments nitrate was reduced for more than 85% to NO as final product by 6 of the 10 *Pseudomonas* strains which normally catalyze complete denitrification to N_2_ (Frunzke and Zumft, 1986). In *P. denitrificans* and *Pseudomonas* SpG59 residual oxygen respiration during anoxic adaptation is required for induction of the denitrification pathway (Kucera et al., 1984; Aida et al., 1986) which was concluded to be a characteristic property of (non-fermentative) facultative denitrifiers (Mazoch et al., 2003).

### Model limitations and outlook

This study demonstrates that the development of a metabolic model improves the level of understanding of the experimental results obtained with *A. tumefaciens* (Bergaust et al., 2008) as it offers a structured look on the processes occurring in the cell. Even though most phenomena could also be identified based on the experimental data, the metabolic model increased understanding. In addition it could be effectively used to identify parameter values (like affinity and inhibition constants), to test hypotheses and will be a good tool in designing new experiments. The model is not intended as a generic metabolic model of the denitrification process.

Extrapolation of the model to the experiment with 1% oxygen gas phase and 1 mM nitrate as initial concentrations revealed that the denitrification rate with nitrate is much lower than with nitrite as electron acceptor. Consequently, a very low nitrate affinity had to be used when using the metabolic model for description of the nitrate experiments when keeping the conversions and enzyme transcription kinetics from nitrite to N_2_O the same. Because the nitrate reduction was slower than the nitrite and NO reduction, the model predicted insignificant accumulation of nitrite and NO. In the experiments however, significant NO accumulation occurred, which can either be due to increased NO production or by decreased NO consumption. As the nitrite reduction rate in the nitrate experiments is already lower than in the nitrite experiments, it is most plausible that the NO reduction is lower than expected based on the nitrite experiments. This can be caused by decreased *norB* transcription, decreased *nor* translation or decreased activity of the NO reductase. Further experiments are needed to identify the exact mechanism.

Currently, not all experiments could be adequately described by the model (see Appendix II). This means that based on the present experimental data-set, some important phenomena could not be identified and additional experiments are needed to improve the model. All experiments described here were batch experiments, characterized by dramatic changes in environmental conditions. Specifically at higher oxygen concentrations the transition from aerobic to anoxic conditions is very fast due to increased biomass concentrations. To which extent these rapid changes affect the overall metabolic state of the cell and the capacity to adjust the metabolism to different conditions remains unclear. To investigate the response of the system to the rates of transition, continuous supply of substrates or products to the experimental system could be applied. Understanding of the regulation network can be further increased by additional measurements of mRNA for known nitrogen oxide sensors and subsequent extension of the metabolic model. Improved parameter estimation could furthermore be established by experiments with external supply of NO, the key intermediate in the denitrification pathway. This may enable improved estimation of inhibition and affinity constants and allow to distinguish better between the effects of nitrite, NO and oxygen limitation.

### Environmental consequences of identified behavior during oxic–anoxic transition

The experimental data of *A. tumefaciens* clearly show that transition from oxic to anoxic conditions can lead to emission of NO. Two circumstances are responsible for increased emission during transition:

- Quick depletion of oxygen; this was found to give rise to incomplete expression of the denitrification pathway and consequently to the increased emission of intermediates,
- Presence of nitrite (already in micromolar range); this resulted in expression of denitrification enzymes and conversion in presence of oxygen.

The characterized independent regulation of *nirK* and *norB* transcription can further increase emission since the expression levels of the individual enzymes can easily be unbalanced.

Dynamic systems that are characterized by rapid transitions from aerobic to anoxic conditions were shown to give rise to increased emissions of NO and N_2_O in practice (Burgess et al., 2002; Kampschreur et al., 2008). NO and N_2_O emissions from oxygen-limiting, nitrite-containing environments have also been observed (Schulthess et al., 1995; Sümer et al., 1995).

Denitrifying organisms are diverse in their regulation of the denitrification pathway (Rodionov et al., 2005), which means that phenomena that were identified for *Agrobacterium* cannot be directly translated towards all facultative denitrifiers. Nevertheless, most phenomena described here have also been identified in other organisms [like *P. denitrificans, R. sphaeriodes* (Baker et al., 1998), *Pseudomonas* (Frunzke and Zumft, 1986), and *Escherichia coli* and *Azotobacter vinelandii* (Borisov et al., 2004)].

### Conflict of interest statement

The authors declare that the research was conducted in the absence of any commercial or financial relationships that could be construed as a potential conflict of interest.

## Figures and Tables

**Figure A1:**
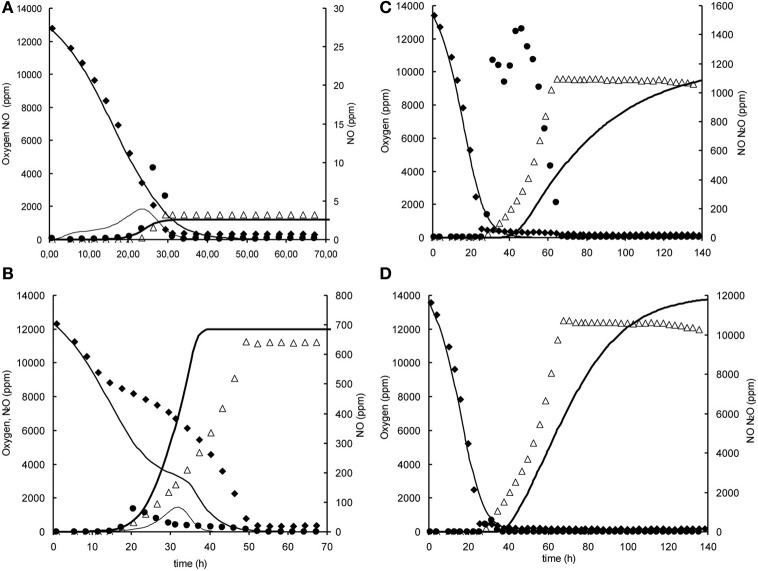
**Modeled (lines) and measured (points) gas phase concentrations of oxygen (♦), NO (•), and N_2_O (∆) during experiments at 1% initial oxygen concentration in headspace with nitrite and nitrate in the liquid phase at 0.2, 1, and 2 mM**. **(A)** 1% oxygen, 0.2 mM nitrite. **(B)** 1% oxygen, 2 mM nitrite, **(C)** 1% oxygen 0.2 mM nitrite. **(D)** 1% oxygen, 2 mM nitrate.

**Figure A2:**
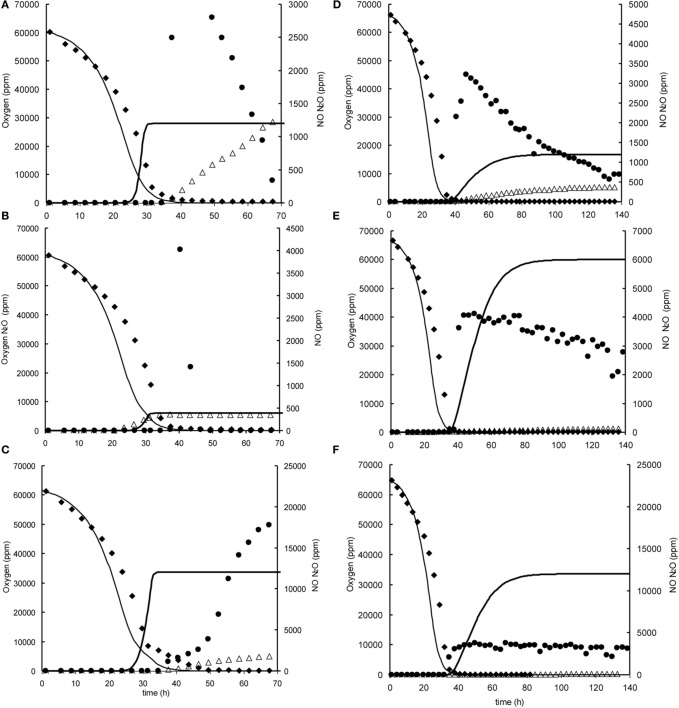
**Modeled (lines) and measured (points) gas phase concentrations of oxygen (♦), NO (•), and N_2_O (∆) during experiments at 7% initial oxygen concentration in headspace with nitrite and nitrate in the liquid phase at 0.2, 1, and 2 mM**. **(A)** 7% oxygen, 0.2 mM nitrite. **(B)** 7% oxygen, 1 mM nitrite, **(C)** 7% oxygen 2 mM nitrite. **(D)** 7% oxygen, 0.2 mM nitrate. **(E)** 7% oxygen, 1 mM nitrate, **(F)** 7% oxygen 2 mM nitrate.

**Figure A3:**
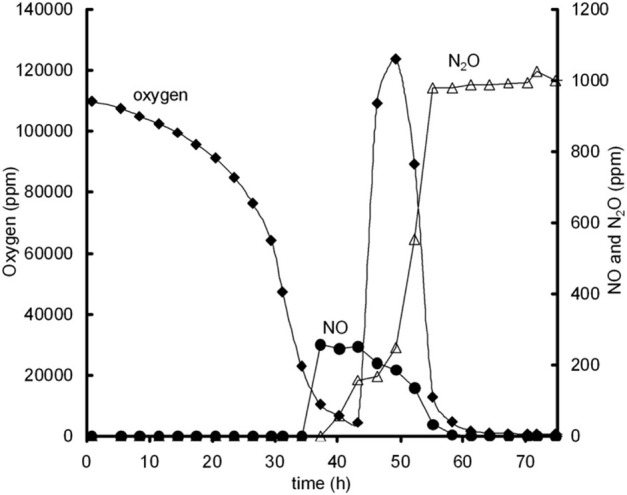
**Oxygen influence on NO reduction activity; oxygen (♦), NO (•), and N_2_O (∆) concentrations during experiment with 13% initial gas phase oxygen and 0.2 mM nitrite**. Oxygen addition is performed after 43 h and leads to immediate increase in NO reduction, as identified from NO decrease and N_2_O increase.

## Appendix I

### Model MATLAB file

function denitrification
close all
clear all
clc

global C_O2m_1mM_1O2 C_COm_1mMni_1O2 Nitrite_1_mM_nitrite_1percent
global NO_1_mM_nitrite_1percent N2O_1_mM_nitrite_1percent
global NIRK_1_1_nitrite NORB_1_1_nitrite
global par k_nir k_nor q_succ K_NO K_NO2 K_NO2_NIR K_NO_NorB K_O2 KI_NO KI_NO_resp
global KI_O2_NIR KI_O2_NOR n_O2_nir

% Kinetic parameters optimized
q_succ     = 0.06565;    % mol S/molX.h
K_NO       = 8.1e-6;     % mM
K_NO2      = 0.88;       % mM
K_NO2_NIR  = 0.0501;     % mM
K_NO_NorB  = 5.42e-5;    % mM
K_O2       = 0.00828;    % mM
KI_NO      = 0.0202;     % mM
KI_NO_resp = 1.74e-5;    % mM
KI_O2_NIR  = 0.00358;    % mM
KI_O2_NOR  = 0.001;      % mM
n_O2_nir   = 3.7;

% Kinetic parameters not optimized
k_nir      = 1/15;       % h-1
k_nor      = 1;          % h-1

par(1) = k_nir;
par(2) = k_nor;
par(3) = q_succ;
par(4) = K_NO;
par(5) = K_NO2;
par(6) = K_NO2_NIR;
par(7) = K_NO_NorB;
par(8) = K_O2;
par(9) = KI_NO;
par(10)= KI_NO_resp;
par(11)= KI_O2_NIR;
par(12)= KI_O2_NOR;
par(13)= n_O2_nir;

% List changing initial conditions in different simulation cases
C0_NO2_case = [0.2    1      2      0.2    1      2     ]; % mM
C0_O2g_case = [0.5406 0.5672 0.5206 2.5423 2.5589 2.5942]; % mmol/l gas
C0_O2l_case = [0.0168 0.0176 0.0162 0.0791 0.0796 0.0807]; % mM

% Load experimental values
C_O2m_1mM_1O2   = load('C_O2m_1mM_1O2.txt');
C_COm_1mMni_1O2 = load('C_COm_1mMni_1O2.txt');
Nitrite_1_mM_nitrite_1percent = load('Nitrite_1_mM_nitrite_1percent.txt');
NO_1_mM_nitrite_1percent      = load('NO_1_mM_nitrite_1percent.txt');
N2O_1_mM_nitrite_1percent     = load('N2O_1_mM_nitrite_1percent.txt');
NIRK_1_1_nitrite = load('NIRK_1_1_nitrite.txt');
NORB_1_1_nitrite = load('NORB_1_1_nitrite.txt');
times=0:80;

% Current simulation case
for ncase=1:6; %for all cases

    % Initial condition (concentrations at time 0)
    % Liquid
    C0_CO2   = 0;
    C0_HCO3  = 0;
    C0_N2O   = 0;
    C0_NO    = 0;
    C0_NO2   = C0_NO2_case(ncase);
    C0_O2    = C0_O2l_case(ncase);
    C0_X     = 0.25;      % mM
    E0_nir   = 0;
    E0_nor   = 0;
    % Gas
    C0_O2g   = C0_O2g_case(ncase);
    C0_NOg   = 0;
    C0_N2Og  = 0;
    C0_CO2g  = 0;

    %            1      2     3     4    5     6    7    8     9     10    11     12     13
    C0 = [C0_CO2 C0_HCO3 C0_N2O C0_NO C0_NO2 C0_O2 C0_X E0_nir E0_nor C0_O2g C0_NOg
	                  C0_N2Og C0_CO2g]';
    options = odeset('RelTol',1e-3,'AbsTol',1e-6);

    [T,Y] = ode23s(...
        @balances,...              % name function containing ODE
        times,...                  % time interval to simulate
        C0,...                     % initial values
        options);                  % options ODE solver

    % printing of model data to text file
    ntimes = length(T);

    fid = fopen(['Simulation_nitrite_final' num2str(ncase) '.txt'], 'wt');
    fprintf(fid, '%15s%15s%15s%15s%15s%15s%15s%15s%15s%15s%15s%15s%15s%15s\n',
    'time', 'C_CO2', 'C_HCO3', 'C_N2O', 'C_NO', 'C_NO2', 'C_O2',
    'C_X', 'E_nir', 'E_nor',
    'C_O2g', 'C_NOg', 'C_N2Og', 'C_CO2g');

    for i=1:ntimes
        fprintf(fid, '%15.5e%15.5e%15.5e%15.5e%15.5e%15.5e%15.5e%15.5e%15.5e%15.5e%15.5e%15.5e
                %15.5e%15.5e\n', T(i),
                Y(i,1),Y(i,2),Y(i,3),Y(i,4),Y(i,5),Y(i,6),
                Y(i,7),Y(i,8),Y(i,9),Y(i,10),Y(i,11),Y(i,12),Y(i,13));
    end
    fclose(fid);
end

end

### Balances MATLAB file

function dcdt = balances(t, y)
global par

k_nir      = par(1);
k_nor      = par(2);
q_succ     = par(3);
K_NO       = par(4);
K_NO2      = par(5);
K_NO2_NIR  = par(6);
K_NO_NorB  = par(7);
K_O2       = par(8);
KI_NO      = par(9);
KI_NO_resp = par(10);
KI_O2_NIR  = par(11);
KI_O2_NOR  = par(12);
n_O2_nir   = par(13);

% Initialize vector of derivatives
dcdt = zeros(length(y),1);   % a column vector

% Transfer vector of variables y in to more friendly variable names
% liquid concentrations
C_CO2 = y(1);   C_HCO3 = y(2);   C_N2O = y(3);    C_NO = y(4);      C_NO2 = y(5);
C_O2 = y(6);    C_X = y(7);      E_nir = y(8);    E_nor = y(9);  % mM
% gas concentrations
C_O2g = y(10);  C_NOg = y(11);   C_N2Og = y(12);  C_CO2g = y(13);

% Initialize net reaction rates for each state variable
r_CO2 = 0;  r_HCO3 = 0;       r_N2O = 0;    r_NO = 0;   r_NO2 = 0;
r_O2 = 0;   r_X = 0;

% Model parameters
% pH
C_H = 3.2e-005; % mM

%q_NO       = 6.28*q_succ*C_X*C_NO/(K_NO+C_NO) * KI_NO/(KI_NO+C_NO) *
              ((KI_O2_NOR/(KI_O2_NOR+C_O2))^3)*E_nor; % mol NO/m3/h
%q_NO2      = 6.28*q_succ*C_X*C_NO2/(K_NO2+C_NO2)*((KI_O2_NIR/(KI_O2_NIR+C_O2))^3)*E_nir;
r_kAB_fast = 1e+09;
pKa_CO2    = 6.36;

% Volumes and mass transfer
V_gas = 0.07; % L
V_liq = 0.05; % L
Vr    = V_liq/V_gas;
kla   = 19.8;     % h-1
H_CO2 = 1.2;    % Mg/Ml
H_N2O = 1.74;   % Mg/Ml
H_NO  = 21.7;   % Mg/Ml
H_O2  = 33;     % Mg/Ml

% Reaction rates
r_hydCO2 = r_kAB_fast*((C_CO2/1000)-(C_HCO3/1000)*(C_H/1000)*10^pKa_CO2);
r_CO2   = r_CO2  - r_hydCO2;
r_HCO3  = r_HCO3 + r_hydCO2;
%------------------------------------------------------------------------
r_groNO = q_succ*C_X*(C_NO/(C_NO*(1+C_NO/KI_NO)+K_NO))^2*KI_O2_NOR/(KI_O2_NOR+C_O2)*E_nor;
Y_groNO_NO  = -6.45;
Y_groNO_N2O = 3.225;
Y_groNO_X   = 1.8;
Y_groNO_CO2 = 2.16;
r_NO    = r_NO    + Y_groNO_NO*r_groNO;
r_N2O   = r_N2O   + Y_groNO_N2O*r_groNO;
r_X     = r_X     + Y_groNO_X*r_groNO;
r_CO2   = r_CO2   + Y_groNO_CO2*r_groNO;
%------------------------------------------------------------------------
r_groNO2 = q_succ*C_X*C_NO2/(K_NO2+C_NO2)*KI_O2_NIR^n_O2_nir/
           (KI_O2_NIR^n_O2_nir+C_O2^n_O2_nir)*E_nir;
Y_groNO2_NO2   = -6.45;
Y_groNO2_NO    = 6.45;
Y_groNO2_X     = 1.8;
Y_groNO2_CO2   = 2.16;
r_NO2   = r_NO2  + Y_groNO2_NO2*r_groNO2;
r_NO    = r_NO   + Y_groNO2_NO*r_groNO2;
r_X     = r_X    + Y_groNO2_X*r_groNO2;
r_CO2   = r_CO2  + Y_groNO2_CO2*r_groNO2;
%------------------------------------------------------------------------
r_groO2 = q_succ*C_X*C_O2/(K_O2*(1+C_NO/KI_NO_resp)+C_O2);
Y_groO2_O2    = −1.19;
Y_groO2_X     = 2.2;
Y_groO2_CO2   = 1.82;
r_O2    = r_O2     + Y_groO2_O2*r_groO2;
r_X     = r_X      + Y_groO2_X*r_groO2;
r_CO2   = r_CO2    + Y_groO2_CO2*r_groO2;
%------------------------------------------------------------------------
r_nir = k_nir*(1-E_nir)*C_NO2/(K_NO2_NIR+C_NO2);
%------------------------------------------------------------------------
r_nor = k_nor*(1-E_nor)*C_NO/(K_NO_NorB+C_NO);

% Gas/liquid transfer rates
rtr_CO2 = kla*(C_CO2g/H_CO2-C_CO2);
rtr_N2O = kla*(C_N2Og/H_N2O-C_N2O);
rtr_NO  = kla*(C_NOg /H_NO -C_NO );
rtr_O2  = kla*(C_O2g /H_O2 -C_O2 );

% Mass balance equations
% CO2
dcdt(1) = r_CO2   + rtr_CO2;
% HCO3
dcdt(2) = r_HCO3;
% N2O
dcdt(3) = r_N2O   + rtr_N2O;
% NO
dcdt(4) = r_NO    + rtr_NO;
% NO2
dcdt(5) = r_NO2;
% O2
dcdt(6) = r_O2    + rtr_O2;
% X
dcdt(7) = r_X;
% E_nir
dcdt(8) = r_nir;
% E_nor
dcdt(9) = r_nor;
% O2 gas
dcdt(10) = - Vr*rtr_O2;
% NO gas
dcdt(11) = - Vr*rtr_NO;
% N2O gas
dcdt(12) = - Vr*rtr_N2O;
% CO2 gas
dcdt(13) = - Vr*rtr_CO2;

end

## Appendix III

### Oxygen influence on NO reduction capacity

Additional experiments were performed in 120 ml serum flasks, containing 50 ml Sistrom's medium (see Bergaust et al., 2008) with 34 mM succinate as the only C source and different concentrations of KNO_2_. The initial oxygen concentration in the headspace was increased to 13% oxygen in the headspace. Inocula of fully dispersed, aerobically grown, late exponential cells of *Agrobacterium tumefaciens*, which had not been previously exposed to anoxic conditions, were injected into the flasks. When oxygen depletion occurred, oxygen was added again to the headspace of the flasks. Figure A3, shows an example of an experiment in which after oxygen addition NO reduction rates increased.
